# Children's Physical Activity, Academic Performance, and Cognitive Functioning: A Systematic Review and Meta-Analysis

**DOI:** 10.3389/fpubh.2020.00307

**Published:** 2020-07-14

**Authors:** Vedrana Sember, Gregor Jurak, Marjeta Kovač, Shawnda A. Morrison, Gregor Starc

**Affiliations:** Laboratory for the Diagnostics of Somatic and Motor Development, Faculty of Sport, University of Ljubljana, Ljubljana, Slovenia

**Keywords:** physical activity, academic performance, teaching qualifications, children, adolescents, intervention

## Abstract

Researching the relationship between physical activity and academic performance is becoming an important research topic due to increasing evidence about the positive effect of physical activity on cognitive functioning. The present systematic review and meta-analysis (PROSPERO registration number: CDR132118) is a unique contribution to the recently published reviews since it only includes interventions longer than 6 weeks and acknowledges the influence of the qualifications of practitioners who deliver interventions. After identifying 14,245 records in five databases and selecting 247 full-text articles assessed for eligibility, 44 interventions passed all eligibility criteria. This meta-analysis uses validity generalization in a random effects model, which shows that academic performance itself is not solely caused by increased physical activity. The weighted mean population effect of all included interventions was r_w_ = 0.181. Most of the studies had serious limitations since they did not report physical activity intensity, which is an essential component to achieving positive exercise effects on cognition. In addition, the qualifications of the staff who administer the interventions were largely ignored in existing literature. It was found that 13 out of 20 physical activity interventions with significant positive effects on academic performance were performed by practitioners who held higher qualifications in the field of physical education and exercise science, who could mediate higher physical activity intensities of the given interventions. The population effect in studies where interventions were administered by practitioners with lower qualifications in the field (r_w_ = 0.14) was lower compared to interventions performed by staff with higher qualifications (r_w_ = 0.22). There was also a significant difference in academic performance with regard to staff qualification level (χ = 4.464; *p* = 0.035). In addition to activity duration, future physical activity intervention studies including those investigating academic performance should focus on the importance of physical activity intensity and include measures of physical fitness as objective indicators to enable more reliable analyses to establish physical activity influence on academic performance.

## Introduction

Regular physical activity (PA) at an adequate intensity and duration is indispensable for maintaining a healthy lifestyle due to its continued positive impact on skeletal (1), metabolic (2), cardiovascular (3) and psychosocial functioning of the human body (4). Low levels of PA, on the other hand, lead to low cardiorespiratory fitness and are associated with a decline in academic performance (AP) (5), possibly due to the deterioration of brain structure, and thus, cognitive abilities and brain function (6–8). PA increases oxygen saturation (9) and angiogenesis (10) in brain areas responsible for task performance. The positive effects of PA on the prefrontal cortex and the hippocampus have been emphasized in many studies (11–13). Furthermore, the molecular architecture and behavior of the basal ganglia may also be directly influenced by PA (8).

Some studies have found positive results between PA and academic performance (AP) (14–17), whereas other studies have found no difference, or even negative correlations between the two factors (18, 19). Review articles using effect size (ES) suggest that PA itself has positive effects on AP (6). For example, one of the earliest meta-analyses in which 1,260 ES were calculated, reported an overall ES of 0.25 and suggested that PA has a small, yet positive effect on cognition (20). ES has been shown to be the largest in studies investigating cognition as a function of fitness level (ES = 0.53). For example, Sibley and Etnier (6) calculated ES for each study that met their study eligibility criteria. They concluded that there is a statistically-significant positive effect of PA on cognition in children, reporting an overall ES of 0.32.

Contemporary children often experience a lack of physical exercise and an abundance of sedentariness in their school and domestic environments, which inevitably leads to deterioration of PA. School-based PA as a part of the normal physical education classes (PE) and extracurricular activities are often the only stable access to PA for many children, and have shown to provide a significant impact on classroom behavior (21), self-esteem (4), self-image (22), and cognitive function (5–7). This is why the competencies of PE teachers and other specialists who deliver school-based PA programmes are so critical, and can affect student outcomes considerably. Formally gained knowledge and professional experiences that assure the professional competencies of the practitioners are key elements for the safe and effective implementation of any PA-enhancing intervention (23, 24). However, the professional competencies of the practitioners who deliver such interventions (and really, any school-based PA programme), are often ignored or not represented in the literature. This meta-analysis does differentiates professional qualifications of staff who perform interventions and measurements in the physical activity classes.

In this regard, our meta-analysis provides a unique contribution to the recently published reviews (25–29), since it exclusively includes interventions longer than 6 weeks (30) and also considers the influence of the qualifications of the PE teachers and practitioners administering the interventions.

Thus, the main purpose of this review is to examine whether primary and secondary school children who were involved in PA-enhancing school-based interventions demonstrated higher AP than their peers who were not involved in regular PA, and whether the improvements observed in AP were influenced by practitioners with higher professional competencies compared to less qualified practitioners. This review examines only those interventions completed in studies with an experimental design, and which reportclear and reliable measures and indicators of both PA and AP. The studies also had to be implemented in school settings.

## Methods

The meta-analysis was performed and reported in accordance with the Preferred Reporting Items for Systematic Reviews and Meta-Analysis (PRISMA) guidelines (31, 32). The present work was registered at the International Prospective Register for Systematic Reviews, identification code CDR132118.

### Literature Search

Two review authors (VS and GS) independently searched literature from databases PubMed, Scopus and ScienceDirect, which were accessed between January 2017 and July 2017 and searches were re-run from February 2019 and June 2019. Gray literature of published interventions and systematic reviews was searched through Google Scholar and Dart electronic databases from February 2019 to June 2019. Unpublished studies won't be sought. The international prospective register of systematic reviews (PROSPERO database) was also searched to identify any unpublished and/or important ongoing meta-analyses. Titles and abstracts of studies retrieved using search string and those from additional sources were screened independently by two review authors to identify potential studies whereas the third review author (GJ) mediated any disagreement until a consensus was reached. Abstracts were screened using following search string: children AND intervention AND school AND (physical activity OR physical education OR extracurricular activity) AND (cognition OR academic performance OR academic achievement). All articles generated from the initial search were stored on Mendeley reference management software & researcher network (Elsevier, Amsterdam, Netherlands) which removed duplicate references.

### Inclusion Criteria

Since the primary objective of the current study was to determine any impact and/or consequences of increased PA on children's behavior and AP, the reviewed interventions had to be published as articles in peer-reviewed scientific journals, as proceedings, or as doctoral dissertations with a focus on healthy school-aged children between 5 and 16 years of age, without disadvantages regarding socio economic standard and with evenly distributed sample in both genders.

The articles also needed to report an obvious measure of increased PA (expressed in minutes per week), including one or more of extracurricular or morning PA, PE, school sports, excluding professional sport activity, including standardized measures of AP (e.g., grade point average, standardized national tests, cognition and intelligence tests, validated tests from algebra, reading and writing).

Only interventions longer than 6 weeks, with control and experimental groups, and with more than 25 participants were included in this review. Results with p < 0.05 were considered statistically significant. All AP outcomes have been recalculated according to the same scale—ES, considering one of the two criteria: (1) ES = 0, no effect; or (2) ES > 0.01, intervention group academically performing better than the control group. Interventions meeting all eligibility criteria are presented in Table 1.

**Table 1:** All interventions included in study.

**Author (year)**	***N***	**Age of children (years or school grade)**	**Intervention duration**	**Experimental design**	**Type of activity and *AP assessment***	**Staff implementing intervention and measurements (HQ or LQ)**	**Intervention min/week**	**Effects on AP (ES)**
Ahamed et al. (18)	288	9–11 years old^**^ (4th and 5th grade)	16 months, 5 days/week; 15 min/day	CG and IG	Increased PA time; *Canadian achievement test*	Classroom teachers (LQ)	47	No effect (0.00)
Alesi et al. (33)	44	8–10 years old	6 months; 2 days/week; 75 min/day	CG and IG	Increased PA time; *Corsi block test, Forward digit span test, The backward digit span test*	PE teacher (HQ)	150	Significantly better executive functions in IG (1.62)
Ardoy et al. (34)	67	12–14 years old (II–V Tanner Grade)	17 weeks, 2 days/week; 55 min/day	CG and 2 IG. (1st IG had only increased PE time, 2nd IG had increased PE time, and intensity of PE lessons)	Enhanced PE and increased PA time*; IGF-M Intelligence Test, and school grades (mathematics, language, natural sciences, English)*	PE teachers (HQ)	110	Significantly better results in mathematics (0.47) and in GPA (2.60)
Beck et al. (35)	165	7.5 (±0.02) years old	6 weeks; 3 days/week; 60 min/day	CG and 2 IG (CG: non-motor enriched mathematical teaching; IG1: fine motor math group; IG2: gross motor math group)	Increased PA time (during academic narrated lessons); *standardized mathematical test, modified Eriksen Flanker test, CANTAB*	Classroom teachers (LQ)	180	No effect (0) after the last testing. Right after the intervention only normal math subgroup in gross motor math IG benefited compared to CG and fine motor IG
Bunketorp Käll et al. (36)	545	12 years old (5th grade)	3 years; 2 days/week; 30–45 min/day	CG and IG	Increased PA time; *Academic performance grades of mathematics, English, and Swedish language*	Not reported (LQ)	75^***^	No effect (0.00)
Chaddock-Heyman et al. (37)	32	8–9 years old	9 months; 5 days/week; 2 school periods/day	CG and IG	Increased PA time; *fMRI*	At university by research staff (HQ)	425^***^	No effect (0.00)
Coe et al. (38)	214	10–11 years old^**^ (6th grade)	1 semester, 5 days/week, On average 19 min of MVPA/day	CG and IG. 1st group was assigned in PE in 1st semester; 2nd group was assigned to PE during 2nd semester	One semester without PE, other semester increased PE time; *Terra Nova Test and individual grades*	Not reported (LQ)	95	No effect (0.00)
Costigan et al. (39)	65	15.8 (±0.6) years old	8 weeks; 1(3)^***^*days/week; 30(120)^****^min/day	CG and 2 IG (CG had normal PE-2 school hours/week; IG had high-interval PE)	Increased PA and enhanced PE; *The trail making test (TMT)*	PE teachers (HQ)	30	No effect (0.00)
Davis et al. (40)	94	7–11 (*M* = 9.2) years old	15 weeks, 5 days/week, 40 min/day	CG (no-exercise) and 2 IG (low-dose exercise group, high-dose exercise group)	Aerobic PA; average heart rate > 150 bpm; *CAS*	PE teachers, researchers (HQ)	200	Significantly better AP in high-dose exercise group (2.24)
Greeff et al. (41)	499	8.1 years old (2nd and 3rd grade)	2 years; 22 weeks/year; 3 days/week; 20–30 min/day	CG and IG	Increased PA time (during academic narrated lessons); *Golden stroop test, Digit Span backward, and Visual span backward test, M-WCST*	Primary and classroom teachers (LQ)	75^***^	No effect (0.00)
Dwyer et al. (14)	~500 in 1st phase (1978); 216 in 2nd phase (1980)	10 years old	14 weeks, 5 days/week, 60 min/day	CG and 2 IG (fitness group and skill programme group)	Enhanced PE time and increased intensity of PA in skill programme group; a*rithmetic and reading test*	Researchers (HQ)	225	No effect (0.00)
Ericsson (42)	251	6–9 years old; (1st, 2nd, and 3rd grades)	3 years, 3 days/week; 45 min/day	CG and 2 IG	Increased PE time (in CG normal curriculum-−2 h per week, in IG 5 h per week); *LUS, national tests in Swedish and mathematics, word, and reading test*	PE teachers (HQ)	135	Significantly better AP in both intervention groups (in national test in mathematics) (0.21)
Ericsson and Karlsson (43)	220	6–9 years old; (1st, 2nd, and 3rd grades) at baseline—follow-up till the 16 years of age	7–9 years, 3 days/week; 45 min/day	CG and IG	Increased PE time (in CG normal curriculum-−2 h per week, in IG 5 h per week); *LUS, national tests in Swedish and mathematics, word, and reading test*	PE teachers (HQ)	135	Significantly better AP in boys IG (1.5) and no effect in IG in girls (0.0)
Erwin et al. (44)	29	8–9 years old (*M* = 8.87) (3rd grade)	20 weeks; 5 days/week; 20 min/day	CG and IG	Increased PA time; *reading and mathematics fluency, school grades, standardized test scores*	Classroom teachers (LQ)	100	Significantly better AP on CBM scores (1, 24) and no effect on standardized tests (0.00) and teachers' grades (0.00)
Fedewa and Davis (45)	460	8–11 years old^**^ (3rd-−5th grade)	8 months; 5 days/week; 20 min/day	CG and IG	Increased PA time (breaks); *Fluid intelligence (SPM), academic performance grades (mathematics and reading)*	Classroom teachers (LQ)	100	No effect (0.00)
Fisher et al. (46)	64	5–7 years old	10 weeks, 120 min/week	CG and IG	Aerobic PA; *CAS, CANTAB, ANT, Conner's Behavior Rating Scale*	Researchers, PE teachers and classroom teachers (HQ)	90	Significantly better AP in intervention group (in ANT and CANTAB and Conner's Behavioral Rating Scale) (0.14)
Gao et al. (47)	208	10–12 years old (*M* = 10, 3 years) (3rd−6th grade)	2 years; 3 days/week; 30 min/day	CG and IG	Increased PA time; *reading and math scores for Utah Criterion Referenced Test*	Classroom teachers (LQ)	90	No effect (0.00); nevertheless greater improvement on math scores of intervention children in Year 1 and 2, the difference was not statistically significant (0.00)
Hedges and Hardin (48)	152	6–7 years old (1st grade)	5 months, 5 days/week, 20 min/week	CG and IG	Increased PA time; *S.A.A.T*	Classroom teachers (LQ)	100	No effect (0.00)
Hillman et al. (49)	221	7–9 years old	9 months; 5 days/week; 2 school periods/day	CG and IG	Increased PA time; *Flanker task, RT, Switch task*	At university by research staff (HQ)	425^***^	Significantly better results in IG in inhibition (0.27), cognitive flexibility (0.35)
Hollar et al. (50)	2,494	6–13 years old	2 years, 5 days/week, 10 min/day	CG and IG	Increased PA and lessons about healthy lifestyle, integrated and replicable nutrition; *FCAT (mathematics and reading)*	Researchers (HQ)	50	Significantly better AP in FCAT math scores (0.21)
Ismail (51)	142	10–12 years old	Academic year, 5 days/week, 60 min/day	CG and IG	Enhanced PE and increased PA with an emphasis on coordination and balance; *S.A.A.T and Otis test*	PE teachers (HQ)	225	Intervention group performed significantly better in AP (0.35)
Kamijo et al. (52)	36	7–9 years old	9 months; 5 days/week; 90 min/day	CG and IG	Increased PA time; *Sternberg cognitive task, EEG activity*	At university by research staff (HQ)	425^***^	Response accuracy was better in IG (0.73), three letter condition was significantly better in IG (0.65)
Katz et al. (53)	352	7–9 years old^**^ (2nd−4th grade)	8 months; 5 days/week; 30 min/day	CG and IG	Increased PA time*; MAP, academic performance grades from communication, mathematics, and arts*	Classroom teachers (LQ)	150	No overall significant change was seen in math AP scores (0.00).
Koutsandreou et al. (54)	71	9–10 years old (3rd and 4th grade)	10 weeks; 3 days/week; 45 min/day	CG and 2IG (the motor-demanding exercise program and cardiovascular exercise program)	Increased PA time; *Letter Digit Span test*	Experienced exercise instructor (HQ)	135	No effect (0.00)
Kvalø et al. (55)	449	10–11 years old (5th grade)	10 months; 5/2 days/week; 20/45 min/day	CG and IG	Increased PA time (during academic narrated lessons, breaks and active homework); *Stroop test, verbal fluency test, digit span, and Trail Making test*	Classroom teachers (LQ)	188	Significantly increased executive function in intervention group (0.21)
Ludyga et al. (56)	36	12–15 years old	8 weeks; 5 days/week; 20 min/day	CG and IG	Increased PA time; *Sternberg cognitive task, EEG activity*	Experienced instructors (HQ)	100	No effect (0.00) in accuracy rates and significant impact on reaction time (0.79)
Mahar et al. (57)	342	5–11 years old	15 weeks, 5 days/week, 10 min/day	CG and IG	Increased PA (during academic narrated lessons); *knowledge test*	Classroom teachers (LQ)	50	No effect (0.00)
Mcclelland et al. (58)	348	7–13 years old	12 weeks; 5 days/week; 20 min/day	CG and IG	Increased PA time; *National examinations in mathematics, reading, and writing*.	Classroom teachers (LQ)	100	IG performed significantly better than CG (0.86) for national exams and (1.24) for progress through National Curriculum levels in reading, maths and writing.
Mullender-Wijnsma et al. (59)	81	8.1 years old (2nd and 3rd grade)	22 weeks; 3 days/week; 20–30 min/day	CG and IG	Increased PA; *Tempo test Rekenen (speed test, arithmetic), Een-Minut Test, Reading*	Classroom teachers (LQ)	75	Mathematics (4.97) and reading (3.38) grades of 3nd grade children were significantly higher and mathematics grade of 2nd grade children were significantly lower (−5.17)
Mullender-Wijnsma et al. (60)	499	8.1 years old (2nd and 3rd grade)	44 weeks, 3 days/week, 45 min/day	CG and IG	Increased PA (during academic narrated lessons); *mathematic speed test and general mathematics skills test, reading, and spelling*	Qualified primary teachers at the beginning of the study, later classroom teachers (LQ)	135	Intervention group perform significantly better in AP: mathematic speed, general mathematics, and spelling (0.43)
Murray et al. (61)	893 (193)	8–11 years old	1.5 years; 5–20 min/day	CG and IG	Increased PA (during academic narrated lessons); *Stanford 10 reading comprehension and math problem-solving achievement tests*	Classroom teachers (LQ)	50^***^	Intervention group perform significantly better in math score and reading (0.31)
Peternelj et al. (62)	134	7–15 years old (1st−8th grade)	8 years; 2 (1st−6th grade) or 1 time/week (7th and 8th grade); 45 min/day	CG and IG	Increased PE time; Academic performance grades (mathematics and language), GPA	PE teachers (HQ)	78, 75^***^	Significant effect only in boys on the language (0.83) and GPA (0.54), whereas no effect in girls (0.00)
Reed et al. (63)	155	7–10 years old	4 months, 3 days/week, 30 min/day	CG and IG	Aerobic PA; *PACT, SPM, Fluid Intelligence Tests*	Researchers and classroom teachers (HQ)	90	Intervention group performed better in Fluid intelligence testing and in PACT tests (0.31)
Resaland et al. (64)	1,129	(*M* = 10.2 years)	8 months;30 min/day (3 times/week); 15 min/day (every day)	CG and IG	Increased PA time (during academic narrated lessons, homework and breaks); *Standard Norwegian national tests (mathematics, reading, English)*	Classroom teachers (LQ)	165	No effect (0.00), some significant effects in subgroups (poorest in the baseline).
Riley et al. (65)	240	10–12 years old (*M* = 11.13) (5th and 6th grade)	6 weeks; 3 days/week; 60 min/day	CG and IG	Increased PA time (during academic narrated lessons); *math performance*	Classroom teachers (LQ)	180	No effect (0.00)
Sallis et al. (19)	655	10–11 years old^**^ (5th and 6th grades)	2 years, 27–42 min/day	CG and 2 IG (group taught by professional PE teachers and group taught by untrained classroom teachers)	Enhanced PE; *Metropolitan achievement tests*	PE teachers and classroom teachers (HQ)	35.5	No effect (0.00)
Shephard et al. (17)	546	6–12 years old^**^ (1st grade-−6th grade)	6 years, 5 days/week, 60 min/day	CG and IG	Increased PA; *Standard provincial tests, teacher grades, WISC test*	PE teachers (HQ)	225	No effect (0.00); intervention group showed some insignificant improvements in AP grades and performed significantly better in Math Provincial Test Scores but significantly worse in overall score and English score.
Sjöwall et al. (66)	470	6–13 years old (1st−6th grade)	2 years; 3 days/week; 60 min/day	CG and IG	Increased PA time; *working memory, arithmetic test*	Activity leader (LQ)	120	No effect (0.00)
Spitzer and Hollmann (67)	44	12–13 years old [*M* = 12,5(IG) and 13 years (CG)] (6th grade)	4 months; 3 days/week; 45 min/day	CG and IG	Increased PA time; *academic performance grades from mathematics, German, and English language*	Classroom teacher (LQ)	135	No effect (0.00)
Tarp et al. (68)	855	12–14 years old (6th and 7th grade)	20 weeks; 5 days/week; 60 min/day	CG and IG	Increased PA time; *Eriksen flanker test for cognitive control, mathematics skills test (algebra, arithmetic, problem-solving, geometry)*	Researchers—external collaborator (also responsible for inviting schools) (HQ)	300	No effect (0.00)
Tuckman and Hinkle (69)	154	9–12 years old	12 weeks, 3 days/week, 30 min/day	CG and IG	Aerobic PA (running); *TDT*	Two experimenters and two undergraduate students (LQ)	90	No effect (0.00)
Niet et al. (70)	112	8–12 years old	22 weeks; 2 days/week; 30 min/day	CG and IG	Increased PA time (break time); *Stroop test, VMS, TMT, ToL*	PE teachers (HQ)	60	No effect (0.00); when taking baseline scores into account, intervention children showed small improvement in Stroop test but no significant differences were found on other executive functioning measures.
Vazou et al. (71)	284	(4th and 5th grade)	8 weeks; 5 days/week; 25–50 min/day	CG and IG	Increased PA time (during academic narrated lessons); *Academic performance grades (mathematics)*	Classroom teachers (LQ)	185	Intervention group performed significantly better in math (0.68)
Zervas et al. (72)	26	11–14 years old	25 weeks, 3 days/week, 75 min/day	CG and IG	Aerobic PA; *Cognitrone test*	PE teachers (HQ)	225	Intervention group performed significantly better in Cognitrone test (2.01)

*Bpa, beats per minute; MVPA, moderate to vigorous PA; CG, control group; IG, intervention group; HQ, staff with higher professional qualifications; LQ, staff with lower professional qualifications; IGF-M, Medium version of the Spanish Overall Factorial Intelligence Test; CAS, Cognitive Assessment Treatment; M-WCST, modified Wisconsin card sorting test; WIAT-II, Wechsler Individual Achievement Test-2nd Edition; LUS, Reading development test; CANTAB, Cambridge Neuropsychological Test Battery; ANT, Attention Network Test; FCAT, Florida Comprehensive Achievement Test; S.A.A.T., Stanford Academic Achievement Test; MAP, Missouri Academic Performance; PACT, Palmetto Achievement Challenge Tests; SPM, Standard Progressive Matrices; WISC, Wechsler Intelligence Scale for Children; TDT, Test of divergent thinking; VMS, Visual Memory Span test; TMT, Trailmakig test; ToT, The tower of London test*.

** age was calculated using national primary and lower secondary school enrolment information;

*** time was calculated using average minutes;

**** *overall intervention time*.

### Data Extraction and Statistical Analysis

One review author extracted data (VS), while the second (GS) and third (GJ) reviewer checked the entered data. Two review authors (VS and GS) extracted outcome data in duplicate, discussing and resolving discrepancies between them and consulted third (GJ) reviewer if necessary. Data supplied for included meta-analysis were checked for missing data and internal data consistency. Summary tables of entered data were checked with the trial protocol and latest trial report or publication. Any discrepancies or unusual patterns were checked with the study investigator. Hunter-Schmidt estimate was used for reducing the amount of bias and Fisher's z transformation was applied to samples' ES to display publication bias (31, 32). We also assessed publication bias with Egger bias test (73).

Chi-square statistical analysis were performed to establish the difference in the effects of interventions on AP in regards to the staff qualifications in PE teaching and exercise science. In the review, we differentiated between staff groups with higher professional qualifications, including exercise science researchers and PE teachers, and staff groups with lower professional qualifications, such as classroom teachers and students, who performed interventions and measurements.

The review includes only studies with enough data to calculate the standardized mean difference (ES) between the intervention and the control group's AP score. For this calculation, the Cohen's (74) and Rosenthal and Rosnow's (75) formulas were used: ES = *M*_1_ − *M*_2_/SD_pooled_ (where SD_pooled_ = √[(${\text{SD}}_{1}^{2}$ + ${\text{SD}}_{2}^{2}$)/2]) and ES = 2t/√*df*), where M_1_ represents the intervention group, M_2_ represents the control group, SD_pooled_ is the pooled standard deviation of both groups, SD_1_ represents the standard deviation of the intervention group, and SD_2_ represents standard deviation of the control group.

In studies that enabled the calculation of more ES, the average ES was used for further analysis. The ES of each intervention was converted to correlation (r_w_) determined by Hunter-Schmidt approach (76), which suggests using pooled within-group SD, because it has less sampling error than the control group. In other words, the aforementioned method corrects ES for measurement error under the condition of equal ES. R_w_ was multiplied by the sample size of each study (r_w_ × N), which represents the numerator and sum of sample sizes represents the denominator of the equation to calculate population effect (r_p_). The generalizability of r_p_ was corrected using an artifact correction and variance sample ES, where the sampling error variance (V_obs_) was based on the population correlation estimate (r_i_) and the average sample size N. The variance due to sampling error was conducted using the equation: V_s_ = (1-${\text{r}}_{\text{w}}^{2}$)^2^/(N-1), where N is the sample size across studies. Estimates of the variance in ES have been calculated using the equation: V_*p*_ = *V*_obs_-V_s_. For weighted mean (r_w_), 95% credibility interval: CI_w_ = r_w_ + 1.96√V_p_ and *I*^2and^ Q statistics to measure heterogeneity of ES were calculated. Descriptors for the used magnitudes of ES were suggested by Cohen (74) and expanded by Sawilowsky (77).

### Assessment of Bias Risk

The assessment of bias risk in the final sample (*n* = 44 studies) was conducted using the criteria previously used by Sneck et al. (78), including the criterion of power calculations. Each study received “0” (does not meet the criterion) or “1” (meets the criterion) based on an analysis of the reporting described in the original article. The Grades Research, Assessment, Development and Evaluation (GRADE) approach was applied to the meta-analysis to determine the quality of evidence; this involved grading the evidence based on a criteria for risk of bias, imprecision, inconsistency, indirectness and publication bias, in conformation with studies reported elsewhere (79, 80).

## Results

**Figure 1:**
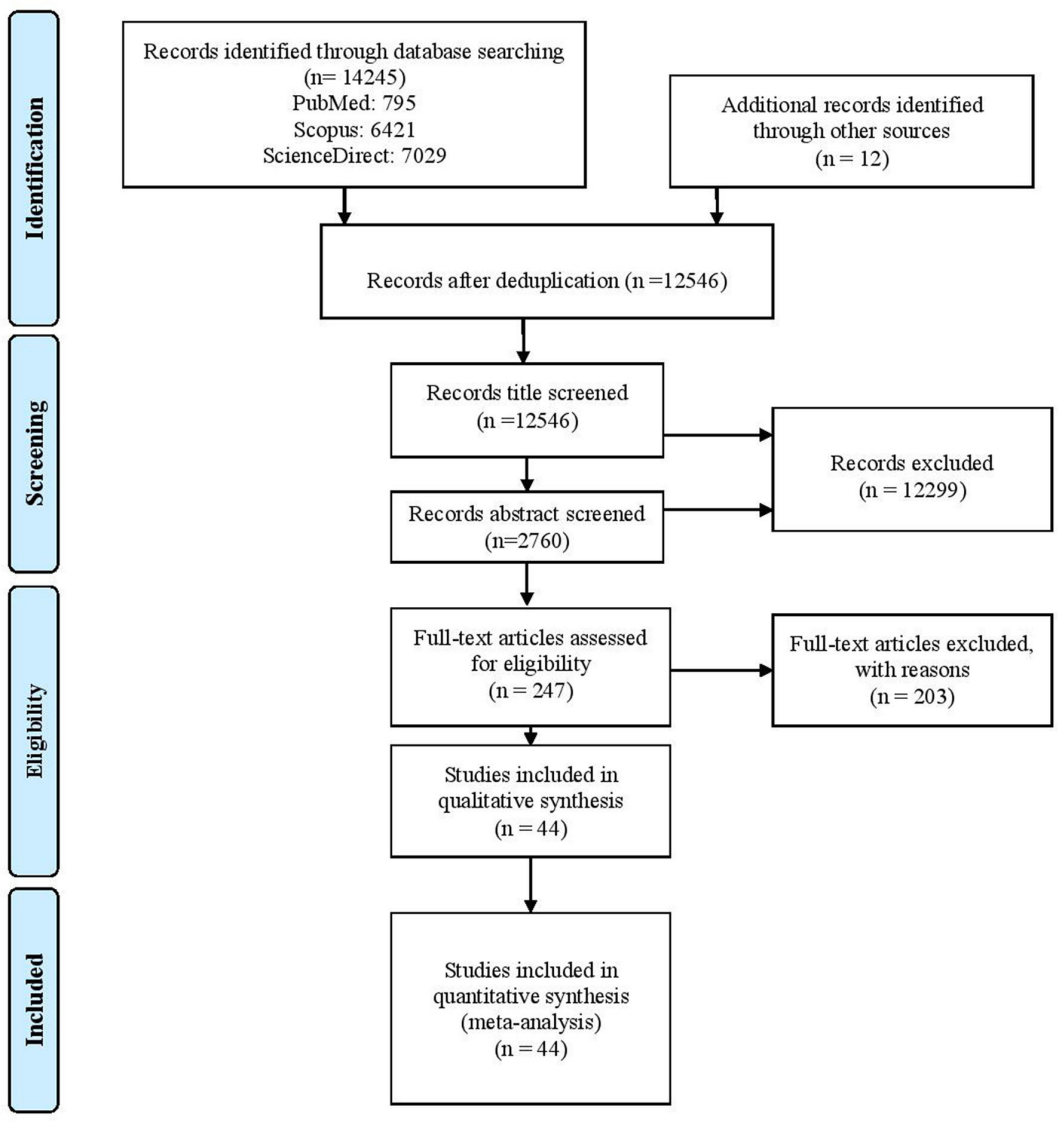
The flow of articles through review.

The flow of the review process is shown in Figure 1. Altogether 14,245 records were identified. After removing duplicates from three different search engines, 591 abstracts with matching key words were identified, and 247 full-text papers were further reviewed. Among them, 68 were intervention studies with the control group. These were thoroughly reviewed, which left 44 intervention studies that met the final criteria for inclusion in the final review.

Altogether, 13,681 children participated in the interventions (*n* = 44) meeting the eligibility criteria. Present review covers over five decades of research studies, ranging from 1967 (51) to 2018 (56). All interventions, meeting all predefined criteria are presented in Table 1.

The mean of intervention time was 60.6 weeks and 144.5 min/week per intervention (Table 2). Duration of intervened time/week in studies with positive effect on AP was 170.9 min/week and 118.3 min/week in studies with no significant effect on AP. Duration of the average intervention time/week was 44.6% longer in studies with positive effect, also, the average study duration (weeks) was 4.9% longer in studies reporting a positive effect compared to studies with none or negative effect of increased PA on AP. Positive results of PA were evidenced in 20 interventions; of this, in 13 studies (65%) the interventions were performed by staff with higher professional qualifications. Negative or null effects on AP were reported in 24 interventions; of these in 9 studies (38%) the intervention(s) were performed by staff with higher professional qualifications (Table 1).

**Table 2:** Descriptive statistics of the main characteristics of interventions included in the review.

	**Number of participants**			**Duration of all interventions (week)**			**Intervention time (min/week)**		
**PA effect on AP**	all	+	–	all	+	–	all	+	–
**Min**	26	26	29	6	8	6	30	50	30
**Max**	2,494	2,494	1,129	411	411	411	425	425	300
**Median**	211	142	215	25	28.5	22	115	135	100
**Mean**	240.5	281.8	294.2	60.6	73.2	69.8	144.5	170.9	118.3

*All, presents all studies included in the meta-analysis; +, studies with positive effect on AP; –, studies with null or negative effect on AP*.

The Hunter-Schmidt method (76) for isolation and correction of sources of error, such as sampling error and reliability of measurement variables, was used. Unweighted mean effect of population is r_u_ = 0.351, whereas weighted mean effect or population effect size is r_w_ = 0.181. This means that groups with increased PA experienced a positive weak effect on AP compared to control groups. Since the variance across sample ES consists of the variance of ES and the sampling error, sampling error variance (V_obs_) for every intervention and variance due to sampling error, using population effect (r_p_), were calculated.

**Figure 2:**
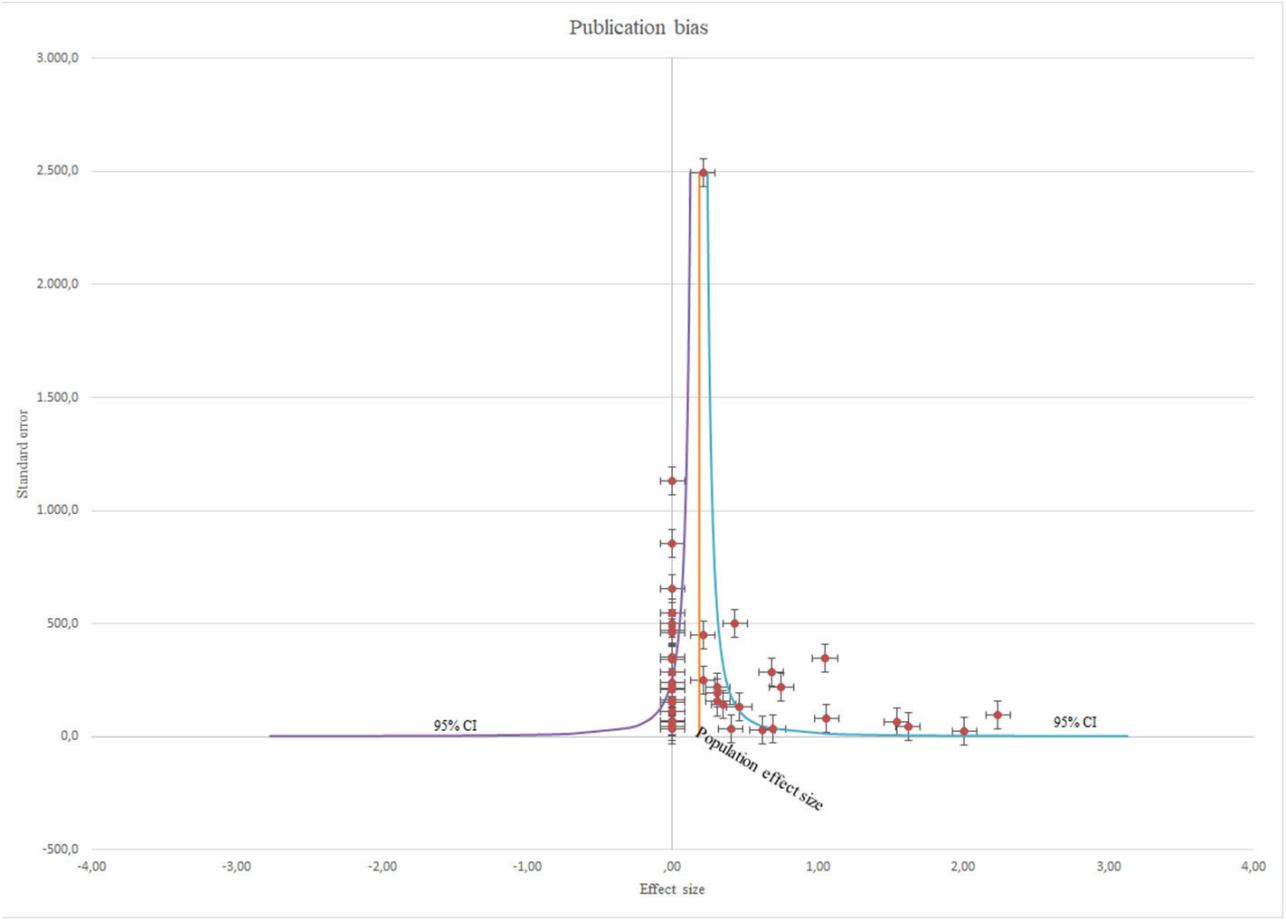
Distribution of ES.

**Figure 3:**
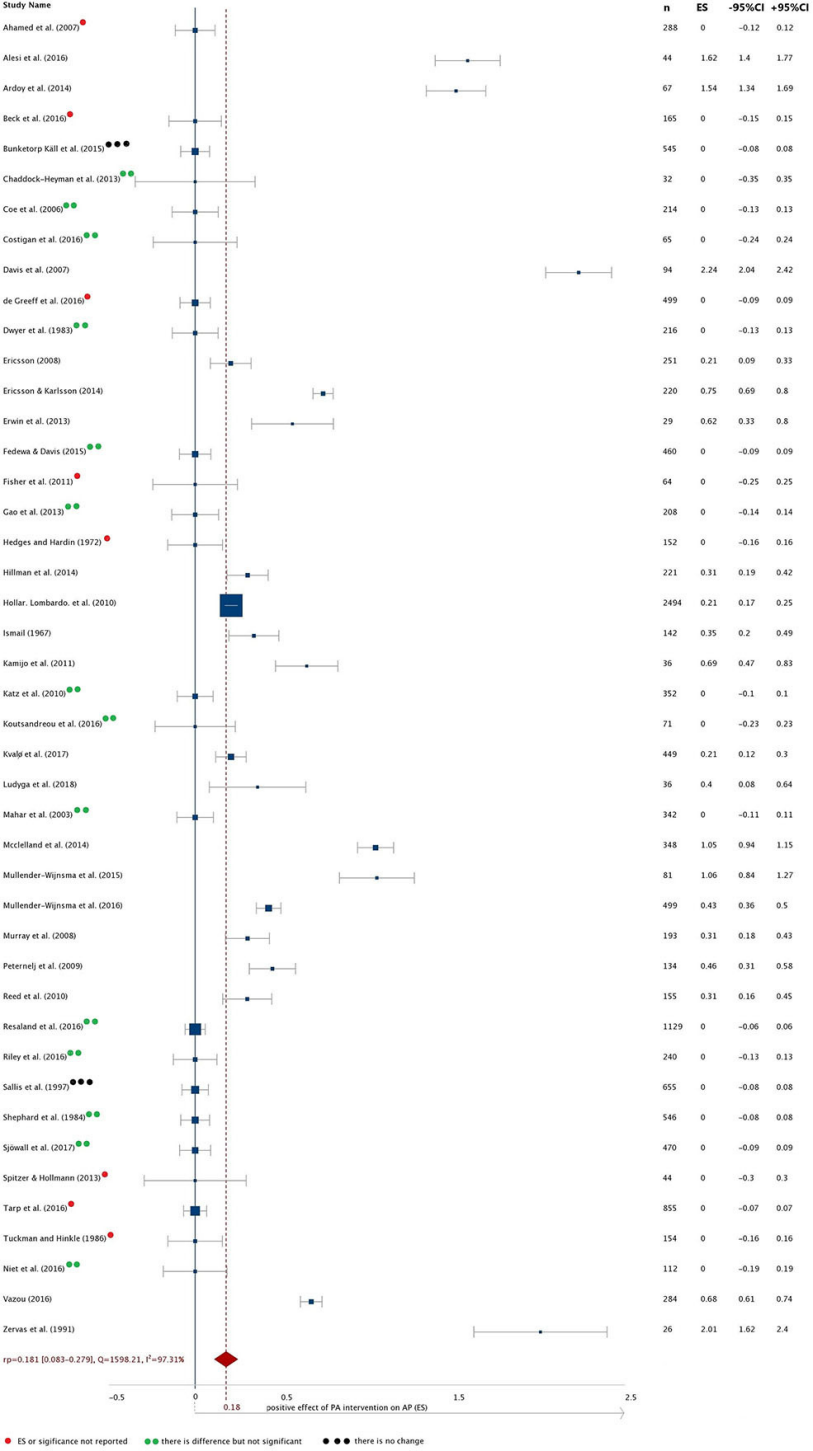
Forest plot of all included studies.

Variance due to sampling error (Vs = 0.003) and variance of population correlations (V_*p*_ = 0.107) were estimated. An average sample size of *N* = 240 yields a population effect size of r_*p*_ = 0.181with 95% confidence interval ranging from 0.083 and 0.279 and 80% credibility interval ranging from −0.237 and 0.600. Such reliable differences between studies that give rise to varying effects could be due to publication bias of included studies (31). The Egger bias test (73) provides significant evidence for publication bias (bias = 4.137, 95% CI: 0.805–7.469, *p* = 0.019). There were considerable differences between the ES's from all individual studies (*I*^2^ = 97%; Q = 1598.2; *p* = 0.000), in studies where staff with higher professional qualifications performed intervention (*I*^2^ = 97%; Q = 970.5; *p* = 0.000) and in studies where staff with lower professional qualifications performed intervention (*I*^2^ = 96%; Q = 622.8; *p* = 0.000). Distribution of ES in all eligible interventions and publication bias (Figure 2), and a forest plot of all included studies (Figure 3) are presented.

The positive effect of a PA intervention on AP was estimated in 13 out of 20 significant interventions in which staff with higher professional qualifications performed the intervention and measurements. The weighted mean effect of the population effect size in interventions, performed by staff with higher professional qualifications, is 0.22 and in interventions performed by staff with lower professional qualifications is 0.14. Chi-square statistics were calculated and showed a significant difference (χ^2^ = 4.464; *p* = 0.035) on AP between studies in which the intervention and measurement were performed by practitioners with higher professional qualifications compared to those conducted by staff with lower professional qualifications, or in studies that lacked this information. Cramer's V value (0.319) showed there is a strong association between staff qualification and its effect on AP (*p* = 0.035).

The results of the risk-of bias assessment analysis are shown in Table 3. Of the 44 studies, 29 were rated as having a low risk of bias (> 67% of total score) with average of 0.79 of total score and 15 were rated as having moderate risk of bias (between 33 and 67% of the total score) with average of 0.50 of total score. None of the studies was rated as having a high risk of bias. Only 11 studies (25 %) reported power calculations to determine sufficient sample size, of those reporting positive effects of PA on academic performance/cognition, power calculations were provided in five studies (34, 46, 49, 55, 60). The quality of evidence (GRADE) where staff with higher and lower qualifications performed intervention and measurements was moderate (Table 4).

**Table 3:** Results of the risk-of bias assessment.

**Author (year)**	**1. Randomization**	**2. Baseline comparable**	**3. Baseline values accounted for in analyses**	**4. Timing**	**5. Blinding of measuring**	**6. Validated outcome measures**	**7. Dropout analysis**	**8. Reporting of results**	**9. Power calculation**	**Total score of the risk of bias (decimal format)**
Ahamed et al. (18)	1	1	1	1	0	1	1	1	0	7/9 (0.78)
Alesi et al. (33)	0	1	1	1	0	0	0	1	0	4/9 (0.44)
Ardoy et al. (34)	1	1	1	1	1	1	1	1	1	9/9 (1.00)
Beck et al. (35)	1	1	1	1	0	1	1	0	0	6/9 (0.67)
Bunketorp Käll et al. (36)	0	0	0	1	0	1	0	1	0	3/9 (0.33)
Chaddock-Heyman et al. (37)	1	1	1	1	1	1	1	1	0	8/9 (0.89)
Coe et al. (38)	1	0	0	1	0	1	1	1	0	5/9 (0.56)
Costigan et al. (39)	1	1	1	1	1	1	0	1	1	8/9 (0.89)
Davis et al. (40)	1	1	1	1	1	1	1	0	0	7/9 (0.78)
Greeff et al. (41)	1	1	1	1	1	1	0	1	1	8/9 (0.89)
Dwyer et al. (14)	1	1	1	1	1	1	0	1	0	7/9 (0.78)
Ericsson (42)	0	1	1	1	0	1	0	1	0	5/9 (0.56)
Ericsson and Karlsson (43)	0	1	1	1	1	1	0	1	0	6/9 (0.67)
Erwin et al. (44)	1	1	1	1	0	1	0	1	0	5/9 (0.56)
Fedewa and Davis (45)	1	0	1	1	0	1	0	1	0	5/9 (0.56)
Fisher et al. (46)	1	1	1	1	1	1	0	1	1	8/9 (0.89)
Gao et al. (47)	0	1	1	1	0	1	0	1	0	5/9 (0.56)
Hedges and Hardin (48)	0	1	1	1	0	0	0	1	0	4/9 (0.44)
Hillman et al. (49)	1	1	1	1	1	1	0	1	1	8/9 (0.89)
Hollar et al. (50)	0	1	1	1	0	1	1	1	0	6/9 (0.67)
Ismail (51)	0	1	1	1	0	0	0	1	0	4/9 (0.44)
Kamijo et al. (52)	1	1	1	1	0	1	0	1	0	6/9 (0.67)
Katz et al. (53)	1	1	1	1	0	1	1	1	0	7/9 (0.78)
Koutsandreou et al. (54)	1	1	1	1	1	1	1	1	0	8/9 (0.89)
Kvalø et al. (55)	1	1	1	1	1	1	1	1	1	9/9 (1.00)
Ludyga et al. (56)	1	1	1	1	0	1	0	1	1	7/9 (0.78)
Mahar et al. (57)	1	1	1	1	1	1	0	1	0	7/9 (0.78)
Mcclelland et al. (58)	0	1	1	1	0	0	0	1	0	4/9 (0.44)
Mullender-Wijnsma et al. (59)	1	0	1	1	0	1	1	1	0	6/9 (0.67)
Mullender-Wijnsma et al. (60)	1	0	1	1	0	1	1	1	1	7/9 (0.78)
Murray et al. (61)	1	1	1	1	0	1	0	1	0	6/9 (0.67)
Peternelj et al. (62)	1	1	1	1	0	1	0	1	0	6/9 (0.67)
Reed et al. (63)	1	0	0	1	1	1	1	1	0	7/9 (0.78)
Resaland et al. (64)	1	1	1	1	0	1	1	1	1	8/9 (0.89)
Riley et al. (65)	1	1	1	1	1	1	1	1	1	9/9 (1.00)
Sallis et al. (19)	1	0	1	0	0	1	1	1	0	5/9 (0.56)
Shephard et al. (17)	0	1	1	1	0	0	0	1	0	4/9 (0.44)
Sjöwall et al. (66)	0	1	1	1	0	0	1	1	0	5/9 (0.56)
Spitzer and Hollmann (67)	0	1	1	1	0	0	0	1	0	4/9 (0.44)
Tarp et al. (68)	1	1	1	1	0	0	1	1	1	7/9 (0.78)
Tuckman and Hinkle (69)	1	1	1	1	0	0	1	1	0	6/9 (0.67)
Niet et al. (70)	0	1	1	1	0	1	0	1	0	5/9 (0.56)
Vazou et al. (71)	0	1	1	1	1	1	1	1	0	7/9 (0.78)
Zervas et al. (72)	1	1	1	1	0	1	0	1	0	6/9 (0.67)
Average of all studies	0.68	0.84	0.93	0.98	0.34	0.82	0.45	0.95	0.25	0.69

**Table 4:** Summary of quality of evidence using the GRADE approach.

**Quality assessment**								**Effect**	**Quality of evidence—GRADE**
**Outcome**	**Study design**	**No. of studies (no. of participants)**	**Risk of bias**	**Inconsistency**	**Indirectness**	**Imprecision**	**Other considerations**	**Rw (95 % CI)**	
Positive effect on AP where staff with higher professional qualifications performed intervention	15 randomized, 7 non-randomized	22 (6,536)	No serious risk of bias (15 low risk of bias, 4 moderate risk of bias).	*No serious inconsistency I*^2^ = 97.8%	No serious indirectness.	No serious limitations.	None	0.22 (0.07–0.37)	⊕⊕⊕□ **MODERATE** (6 high, 13 moderate, 3 low)
Positive effect on AP where staff with lower professional qualifications performed intervention	15 randomized, 7 non-randomized	22 (7,145)	No serious risk of bias (2 moderate risk of bias, 5 high risk of bias).	*No serious inconsistency I*^2^ = 96.6%	No serious indirectness.	No serious limitations.	None	0.14 (0.02–0.27)	⊕⊕⊕□ **MODERATE** (4 high, 14 moderate, 4 low)

*GRADE, Grades of Research, Assessment, Development and Evaluation (GRADE Working Group)*.

*⊕⊕⊕⊕ (high): We have a lot of confidence that the true effect is similar to the estimated effect*.

*⊕⊕⊕□ (moderate): We believe that the true effect is probably close to the estimated effect*.

⊕⊕□□ (low): We believe that the true effect might be markedly different from the estimated effect

*⊕□□□ (very low): We believe that the true effect is probably markedly different from the estimated effect*.

## Discussion

This systematic review and meta-analysis investigated the impact of school-based PA interventions on the AP of primary and lower- secondary schoolchildren; it considered the amount of PA in the interventions and the qualifications of staff administering interventions in its analysis. The main findings of the study are that changes in AP itself is not caused solely by an increase in frequency and/or duration of PA, but studies must also take into consideration the intensity of PA administered. Secondly, and of equal import, is the significant positive effect observed when PA interventions are delivered by practitioners with higher professional qualifications, who were able to mediate higher PA intensity and focus of interventions.

Despite some promising results in the reviewed interventions (31, 33, 34, 37, 40, 42, 46, 49, 50, 52–55, 57, 58, 60, 61, 63, 71, 72), it is essential to emphasize that future research on the relationships between AP and PA should consider more qualitative aspects of PA, including intensity and types of activities. Namely, positive effects of AP may only accrue when of moderate-to-vigorous PA is increased (41, 42). Although the effects of PA on AP are seemingly well-documented in the literature most research and review studies have focused on the behavioral aspects of PA, such as frequency and duration, whereas PA intensity was hardly addressed, even though it is essential for properly elucidating the effects of exercise on cognition (43).

To avoid reporting sometimes misleading results of short interventions that are often affected by an initial increase in motivation of study participants (i.e., researchers, teachers, parents and children), only interventions longer than 6 weeks' duration were included for this analysis. The analysis showed that in the longer-lasting interventions there was a greater decline in moderate-to vigorous PA across the intervention time.

Samples in interventions which reported positive PA impacts were larger than the ones in the interventions with negative or no impact; this should be taken into consideration when interpreting the results. The population effect (r_p_) was shown to be small, which means that increased PA in experimental groups had only a small effect on AP compared to control groups. Since the connection between both variables was small, PA alone may not be the best predictor of children's AP. When researching the relationships between cognition and activity, researchers should start thinking about utilizing and reporting more stable measures, like the level of physical fitness, which is a marker outcome of habitual physical activity. For example, it has been shown that after-school PA improves cardiovascular endurance in children, which then mediates improvements in AP (81). A meta-analysis of published evidence on the relationship between physical fitness and AP between 2005 and 2015 asserts that cardiorespiratory fitness, speed-agility, motor coordination, and perceptual-motor skills are highly associated with AP (45), but the findings on the relationship between AP to strength and flexibility remain unclear in this regard. Indeed, it can be argued that physical fitness may be a better predictor of AP than PA. Several studies have identified a positive relationship between cardiorespiratory fitness, weight and AP (46–49) or with overall physical fitness (82). For example, researchers from Portugal have shown that cardiorespiratory fitness is independently related to AP; moreover, students with normal weight tended to have the best academic performance (52). The influence of moderators and mediators to PA, such as socioeconomic status, parental education (83), concentration (84) gender (85), personality (86), motivation, body-image, self-esteem (87), have also been shown to have an impact on AP.

In almost all analyzed interventions, the higher qualifications of the involved staff were shown to positively influence changes observed in AP. We know that PE lessons led by trained PE teachers provides more activity to students than ones led by generalized classroom teachers (25, 88–90). In general, the pedagogical qualifications of classroom teachers for delivering PE classes are far lower than those for of specialist PE teachers. The classroom teachers are of course qualified teachers, but with a rather limited training in PE teaching and exercise science, which requires specialist training to perform well. They often experience insufficient expertise and ability to organize PE with its distinctive content in an effective way (91). Breslin et al. (92) showed that the PE specialists show higher levels of self-determination toward exercise, are more autonomous in their decisions to be active, are more physically active and have a higher level of perceived qualification in delivering a PE lesson than the generalist teachers do. In addition, McKenzie et al. (89) reported that children taught by PE specialists spend 57% more time in moderate-to vigorous PA, with a concurrently increased emphasis on the promotion of physical fitness. This also has the important advantage of maintaining positive health-related behaviors that may last beyond childhood (93, 94).

### Study Strengths and Limitations

The present review covers more than five decades worth of research studies, but analyses only PA interventions that have met the predefined criteria. Therefore, only interventions published in peer-reviewed scientific journals with a focus on primary and lower secondary school-children, evenly represented genders, exceeding 6 weeks and with reported ES or with data that enables the calculation of ES were included in the meta-analysis. The analysis did not: (i) distinguish between the published results that used moderate-to vigorous PA as a significant predictor that might affect AP and the ones that used low or vigorous physical activity; (ii) it did not take into consideration the differences between the results deriving from subjective or objective measures of PA; (iii) it did not take into consideration different instruments for assessing AP that were used in various studies, thus results cannot be generalized to different measures of AP; (iv) it did not take into consideration the differences between studies with large sample size that were able to detect even small differences and the ones with smaller sample sizes that could not, which can lead to potential bias; and it should always be noted that (v) statistically non-significant results are less-likely to be published, resulting in upwardly biased meta-analytically derived effect sizes for any analysis of this kind.

## Conclusion

Parents are often concerned that time allocated to PA and sport may negatively affect children's AP. The present analysis shows that PA itself does not negatively affect AP; moreover, there are positive, (although relatively small) relationships between the two, and that changes in AP itself is not caused solely by an increase in frequency and/or duration of PA, but studies must also take into consideration the intensity of PA administered. Secondly, the significant positive effect of PA interventions are most observed when delivered by practitioners with higher professional qualifications who are able to mediate higher PA intensity in the interventions. Finally, in interventions with long durations, there are greater declines in moderate-to vigorous PA, suggesting a challenge to maintaining interventions across the intervention time-span. Finally, when reporting the monitoring, surveillance and evaluation of PA interventions, using physical fitness as criteria measure of PA is much more effective in terms of both the economic and organizational sense.

## Data Availability Statement

All datasets generated for this study are included in the article/supplementary material.

## Author Contributions

All authors listed have made a substantial, direct and intellectual contribution to the work, and approved it for publication.

## Conflict of Interest

The authors declare that the research was conducted in the absence of any commercial or financial relationships that could be construed as a potential conflict of interest.
